# Effects of residual PAH exposure from firefighters’ skin and turnout gear on biospecimen microRNA expression

**DOI:** 10.1016/j.envres.2025.122348

**Published:** 2025-07-12

**Authors:** Jooyeon Hwang, Jenny R. Gipson, Chao Xu, Timothy VanWagoner, Xin Xu, Robert J. Agnew, Willard M. Freeman

**Affiliations:** a Department of Environmental and Occupational Health Sciences, School of Public Health, University of Texas Health Science Center at Houston, Houston, TX, USA; b Southwest Center for Occupational and Environmental Health, University of Texas Health Science Center at Houston, Houston, TX, USA; c Laboratory for Molecular Biology and Cytometry Research, The University of Oklahoma Health Sciences, Oklahoma City, OK, USA; d Department of Biostatistics and Epidemiology, Hudson College of Public Health, The University of Oklahoma Health Sciences, Oklahoma City, OK, USA; e Oklahoma Clinical and Translational Science Institute, University of Oklahoma Health Sciences, Oklahoma City, OK, USA; f Shanghai Anti-doping Laboratory, Shanghai University of Sport, Shanghai, China; g Fire Protection & Safety Engineering Technology Program, College of Engineering, Architecture and Technology, Oklahoma State University, Stillwater, OK, USA; h Genes & Human Disease Research Program, Oklahoma Medical Research Foundation, Oklahoma City, OK, USA; i Oklahoma City Veterans Affairs Medical Center, Oklahoma City, OK, USA

**Keywords:** Firefighter, Fire smoke, Polycyclic aromatic hydrocarbons, Biospecimens, Turnout gear, MicroRNA expression, Transcriptome

## Abstract

Firefighters are routinely exposed to polycyclic aromatic hydrocarbons (PAHs) during fire suppression activities, molecular biomarkers reflecting such exposure remain underexplored. Thus, we explored an integrated exposure assessment framework linking PAH contamination on turnout gear and skin to changes in microRNA (miRNA) expression in skin and blood biospecimens. Our goals are to evaluate the relationship between fire-related PAH exposure and miRNA expression profiles in firefighters, and to identify candidate miRNA biomarkers of occupational exposure. This prospective study enrolled 25 firefighters. PAHs on turnout gear and skin were quantified post-fire using gas chromatography/mass spectrometry. Concurrently, skin tape strips and blood were collected and analyzed for miRNA expression using NanoString’s nCounter platform. Bioinformatic analyses, including differential expression, pathway enrichment, and network modeling, were performed to evaluate PAH-associated miRNA dynamics. Five miRNAs showed significant differential expression following fire activity (q < 0.05), with miR-125a-3p showing a 60 % increase post-fire. Biospecimen comparisons showed six differentially expressed miRNAs, including a 91 % reduction in miR-451a expression in skin relative to blood. Network and pathway analyses identified PAH-specific interaction patterns, with Naphthalene emerging as a central node. Pathway enrichment highlighted necroptosis and Th17 cell differentiation as key biological processes affected by exposure. This study provides novel evidence that PAH exposure at fire scenes induces distinct miRNA expression profiles in firefighters. miR-125a-3p emerges as a potential biomarker responsive to fire-related exposures. These findings provide critical insights into PAH-associated transcriptional responses and suggest the potential of miRNA profiling in occupational exposure assessment and cancer risk stratification among firefighters.

## 1. Introduction

Firefighters are covered with smoke-derived organic compounds from fire-related activities and their turnout gear (Alexander and Baxter, 2016; IARC, 2010; Kirk and Logan, 2015; Wingfors et al., 2018). Accordingly, the byproducts of combustion detected at fires have been elaborated as Group 2B (possibly carcinogenic) to Group 1 (carcinogenic) occupational exposures by the International Agency for Research on Cancer (IARC), (Demers et al., 2022). Of these fire-related contaminants, polycyclic aromatic hydrocarbons (PAH) are the most commonly studied and best understood carcinogenic substances produced during firefighting activities (Baris et al., 2001; Dahm et al., 2015; Fabian et al., 2014). PAH, frequently cited for their mutagenic and teratogenic potential, can damage human DNA (Abdel-Shafy and Mansour, 2015; Fent et al., 2014; Kim et al., 2013; Moen and Ovrebo, 1997; Rengarajan et al., 2015). This damage can result in chromosome abnormality and genetic instability, potentially leading to cancer (Deng et al., 2014). Firefighters are exposed to PAH through smoke inhalation during a fire response or off-gassing after a fire activity (Hwang et al., 2019a). Similarly, they are exposed through skin absorption of accumulated PAH when donning or doffing contaminated personal protective gear (PPE). Consequently, the PAH dose on firefighters skin has been found to be positively correlated with DNA strand-break levels in peripheral blood mononuclear cells (Andersen et al., 2018).

Researchers recognize that the incorporation of human miRNA profiles into exposure assessments lead to a better understanding of chronic diseases like cancer (Rappaport and Smith, 2010). An miRNA is a small, endogenous, single-stranded, noncoding RNA that regulates expression by targeting messenger RNA (mRNA), (Ambros, 2004). The alteration of miRNA by occupational exposures at a fire activity can increase deleterious health effects by influencing gene expression or stability. Thus, an understanding of miRNA and altered transcriptomic signatures/pathways inside the body reveal important biomarkers relevant for assessing stress, short-term risks, and long-term consequences. In particular, miRNA, which characterizes individual susceptibility, may be used to measure candidate biomarkers of exposure to PAH and develop a statistical model of the causal association for firefighters. In vivo and in vitro miRNA with PAH in human cell studies detailing blood or other target tissues has been reported to induce gene promoter hypomethylation, which can increase the risk of cancer (Tilton et al., 2015; Yamamoto et al., 2013). To date, however, the mechanism underlying the PAH-associated carcinogenicity of residual exposures on turnout gear from fire-related activities has not been fully understood.

Previous studies have demonstrated that exposure to hazardous environmental and occupational factors may significantly disrupt miRNA expression patterns. Most of those studies were environmental or assessed genome-wide changes in miRNA expression profiling due to particulate matter in ambient air (Fossati et al., 2014; Fry et al., 2014; Motta et al., 2013). Only limited studies on PAH-associated miRNA have been conducted in occupational settings. Deng’s group found dysregulated miRNA (miR-24–3p, miR-27–3p, miR-142–5, miR-28-5p, and miR-150–5p), (Deng et al., 2014). which are associated with oxidative DNA damage levels, in plasma from PAH-exposed coke-oven workers. In addition, they discovered that four of the PAH-associated miRNAs (miR-24-3p, miR-27-3p, miR-142–5, and miR-320b) were negatively associated with heart rate variability indices, which indicates that these miRNAs are involved in the regulation of cardiac autonomic functions or the cardiovascular system (Huang et al., 2016). Similarly, women exposed to PAHs from biomass smoke had significantly higher plasma miR-126 and miR-155, both linked to cardiovascular disease (Ruiz-Vera et al., 2019). A subset of a firefighter cancer prevention study found that eight miRNA expressions (miR-548h-5p, miR-145–5p, miR-4516, miR-331–3p, miR-181a-5p, miR-1260a, miR-374a-5p, miR-486–3p) associated with cancer pathways differentiated between incumbent and new firefighters (Jeong et al., 2018). The follow-up longitudinal study highlighted a significant association between specific miRNAs and employment duration, structural fire exposure (fire-hours and fire-runs), and time since the most recent structural fire. Notably, miR-494–3p and miR-422a were significantly downregulated with longer employment duration, while miR-548ad-3p was upregulated, and all have been linked to various cancer pathways. These findings suggest that these miRNAs could serve as biomarkers for cumulative exposure and increased cancer risks in firefighters. However, only general demographic characteristics or surrogate exposure to fire smoke, not actual measurements of fire smoke concentrations, were considered in any of these studies.

This study presents a novel exposure assessment strategy for synthesizing the gene and transcriptomic alterations as biomarkers for occupationally exposed firefighters. We demonstrate a systematic approach to associating residual exposures to PAH on turnout gear and skin with dermal and blood miRNA expression among firefighters. Specifically, we examine the direct impact of fire-smoke exposure on firefighters’ miRNA expression in dermal and blood samples, identifying the PAH-associated profiling and alteration of miRNA expression by comparing pre- and post-fire-related activities.

## 2. Subjects and methods

### 2.1. Study design

The design of this study has been described in detail in our earlier publication (Zhu et al., 2024). In brief, pre- and post-exposure assessments were conducted over a period of six months (Dec. 2021–Jun. 2022) to assess the biological effects of exposure to PAH from fire smoke on miRNA expression in biospecimens. Orientation days were scheduled to visit fire departments and conduct pre-exposure sampling campaigns, explaining the purpose of the study and the general procedures to the potential participants. After their eligibility was confirmed, participants signed an informed consent form approved by the Institutional Review Board (IRB #11466) of the University of Oklahoma Health Sciences. Baseline demographic characteristics, turnout gear practices, and firefighting-related practices were recorded via self-administered questionnaires. Unlike a traditional pre-post study design, we began to conduct post-exposure sampling campaigns due to the unpredictability of fire events. For baseline pre-exposure sampling, we revisited each participating fire department no earlier than 24 h (1 day) after the fire activity. The interval between the post- and pre-sampling campaigns ranged from 1 to 90 days.

### 2.2. Study participants

The recruitment of study participants was a collaborative effort between local and regional fire departments in Oklahoma because groups of career and volunteer firefighters from different departments make runs together to a fire scene. These fire departments are located within a 100-km radius of the institutional field team to facilitate timely responses, as longer distances would cause delays. To be eligible, a firefighter had to be 18 years of age or older, with at least one year of experience subsequent to probation, and possess their own turnout gear, as PAH on their gear and related information on their gear practices were collected. To maximize control of the background PAH levels, firefighters were excluded who 1) used any tobacco products; 2) had eaten grilled, barbecued, or smoked foods within two days of the sampling collection; 3) had a history of chronic/infectious diseases; or 4) used medications. As a further precaution, volunteer firefighters who had potentially been exposed to PAH in a primary occupation were excluded.

### 2.3. Environmental PAH and biospecimen collection

The procedure for collecting PAH samples from firefighters has been previously described in detail in (Zhu et al., 2024). In brief, PAH deposited on the surface of firefighters’ turnout gear and skin were collected using a 100 cm^2^ polyester fabric wipe (Alpha Wipes, Texwipe, Kernersville, NC) saturated with 1 ml of 99 % isopropyl. The collected wipes from the turnout gear and skin were analyzed by gas chromatography/mass spectrometry using EPA Method 8270D. For quality control, at least one field blank, without wiping, was collected for every ten samples.

Skin specimens were collected from firefighters using a skin tape strip (STS, D-Squame discs, 1.4 cm diameter, CuDerm, Dallas, TX). The tape was applied to the skin 20 times all around the wrist and neck areas opposite the environmental PAH skin wipe spots, with pressure equivalent to 225 g/cm^2^ (D500 Pressure Instrument, CuDerm, Dallas, TX) for 3 s each time (Dyjack et al., 2018). The STS specimens were stored in microfuge tubes at −80 °C until further analysis. Blood samples were collected by a certified phlebotomist from the Oklahoma Shared Clinical and Translational Resources (OSCTR) at the University of Oklahoma Health Sciences. At the field site, blood samples were drawn in EDTA-containing tubes to avoid coagulation and processed into serum by centrifuging per standard operating procedure (World Health Organization, 2010). All of the collected biospecimens were transported on ice to the Laboratory for Molecular Biology and Cytometry Research (LMBCR) at the University of Oklahoma Health Sciences on the same day and stored at −80 °C for further analysis.

### 2.4. Laboratory analysis

#### RNA extraction:

RNeasy Micro Kits (Qiagen, Chatsworth, CA) were used to isolate RNA from the STS and serum samples according to the manufacturer’s protocol. The STSs were placed on a sterile, flat surface, adhesive side up. While the STSs were held in place with forceps, sterile #10 blades were used to scrape the STS surface. The blades were dipped into sterile, LoBind tubes containing 350ul RLT Buffer+β-mercaptoethanol (Qiagen, Chatsworth, CA). Since the adhesive material was also scrapped off, a sterile pipet tip was used to remove the sample from the blade and into the buffer. Dipping the blade in the RLT buffer helped with scraping the material from the tape. Any adhesive was trapped in the column during purification, preventing downstream issues. 200 μL of thawed serum were also transferred to sterile, LoBind 2 ml tubes for RNA isolation. RNA was isolated using Qiagen’s miRNeasy Serum/Plasma Advanced Kit and established protocols. Upon completion, the samples were then concentrated using RNA Clean & Concentrator-5 (DNase Included) (Zymo Research, Irvine, CA).

#### miRNA expression analysis:

The RNA yield for both sample types was too low to quantify using Qubit RNA HS Assay Kits or Agilent Bioanalyzer RNA system. Therefore, the maximum 3 μL of RNA was deposited into the nCounter miRNA Expression Assay Kit (#CSO-MIR3–12, Human v3 miRNA Assay, NanoString, Seattle, Washington) provided with the panels. Its high sensitivity allowed us to detect miRNAs that had been significantly altered by environmental exposures. The quantity and quality of miRNA were assessed using the NanoDrop ND-1000 Spectrophotometer (Thermo Fisher Scientific, Wilmington, DE) and the Agilent 2100 Bioanalyzer (Agilent Technologies, Santa Clara, CA). The expression levels of miRNA were ligated with bridge oligos and miRNA tags, making them long enough to hybridize to the probes using the nCounter Human v3 miRNA Expression panel with 800 endogenous human-associated miRNAs (NanoString Technologies, Seattle, WA). Prepared miRNA samples were then hybridized with the capture and reporter probes for 16 h at 65 °C. Immediately after hybridization, the samples were loaded onto the NanoString nCounter Max Analysis System for clean-up and detection. Each run contained miRNA from six skin samples and six serum samples.

#### Data processing:

The raw data were logarithm 2-transformed and then normalized as the manufacturer suggests (Hou et al., 2016; Rodosthenous et al., 2016). The RCC and RLF files were imported into NanoString’s nSolver Advanced Analysis Software v 4.0. Analysis of the normalized data was performed with the background correction threshold count value set to 30, no positive control correction, and normalization by geometric mean set at the top 100 genes. The normalization file was then imported into ROSALIND and processed through the miRNA nSolver normalized counts pipeline.

#### Bioinformatic analysis:

The limma R package was used to calculate fold changes and p-values and perform optional covariate correction. Clustering of miRNA for the final heatmap of differentially expressed miRNA was performed using the heatmap R package. The top targeted gene predictions, validated genes, and related drugs and diseases were analyzed using the multiMiR R package. miRNA secondary structures were calculated and visualized using ViennaRNA software. The paired miRNA sequencing data were analyzed using DESeq2 R package. We modeled sample type (skin versus blood) and exposure status (preversus post-fire activity), simultaneously, controlling for the top two principal components to adjust for unobserved confounders and batch effects. We also used a linear mixed model to analyze the association between PAH level as a predictor and miRNA data as the outcome variable from repeated measurements, adjusting for possible confounders based on the questionnaire. Stratified analysis by sampling type of miRNA and PAH were conducted separately. P-value was adjusted based on false discovery rate (FDR). All reported adjusted p-value (q-value) < 0.05 was the significance level. Based on the Pearson correlation and agreement analysis, we compared the expression profiles of miRNAs, especially the newly identified exposure-associated miRNAs, from the skin and blood samples.

#### Functional and pathway analyses:

A computationally predicted and experimentally validated database, including the University of California Santa Cruz’s (UCSC) Genome Browser was used as the primary source for the gene targets of differentially expressed miRNAs. The database was used to detail pathway, gene ontology, organ, and other categories (Hou et al., 2016). In addition, the Kyoto Encyclopedia of Genes and Genomes (KEGG) database was used to conduct an analysis of functions, such as oxidative stress and immune/inflammatory processes enrichment (Bollati et al., 2010). Hierarchical clustering and a heatmap were used to identify the shared miRNAs between sample types, as well as to assess the strength of the association between each miRNA and the relevant pathway. We used the QIAGEN Ingenuity Pathway Analysis (IPA) to conduct the network, pathway, and functional analyses (Krämer et al., 2014). describes the algorithms developed for use of IPA.

## 3. Results

Twenty-five firefighters recruited from five fire departments in Oklahoma were followed for blood and skin samples before and after fire activities. Table 1 shows no statistically significant demographic differences between career and volunteer participants (p > 0.05). Given the similar demographic characteristics and work practices (Table 1), one plausible reason is that volunteer and career firefighters are exposed to similar levels of PAHs regardless of exposure patterns. For example, volunteer firefighters lack the resources needed to properly maintain, clean, and store their turnout gear. Career firefighters deal with more frequent exposures to fire-related contaminants during training and while on duty (Hwang et al., 2019a, 2019b). As a result, there were no significant differences between the two groups in the levels of accumulated PAHs on gear and skin, except for Acenaphthene (Zhu et al., 2024). This result aligns with our previous study on firefighter vehicles. The residual PAH concentrations for all analytes in volunteer firefighters’ vehicles showed no statistically significant differences from the career firefighters’ trucks, suggesting similar contaminant exposure potentials for the two groups (Hwang et al., 2019a). The participants’ average age was 39 years, with career firefighters being slightly younger (36 years) compared to volunteers (41 years). Both groups had an average of 12 years of service as a firefighter. Most firefighters were male (92 %) and predominantly non-Hispanic White (96 %). At an average height of 1.8 m, career firefighters weighed 89 kg (BMI: 27.5, overweight), while volunteers weighed 99 kg (BMI: 30.6, obese). The demographic similarities suggest combining both groups for further analysis.

**Fig. 1.**
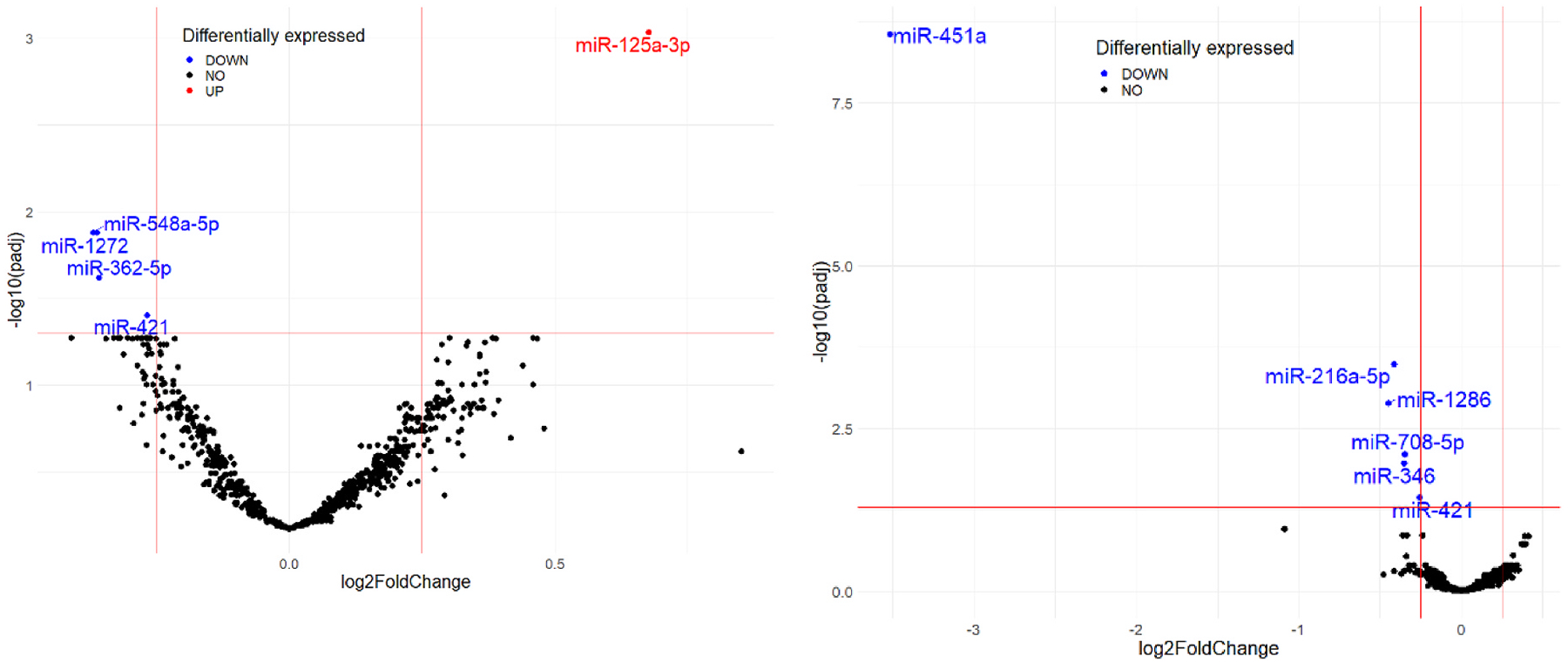
Volcano plot of Post vs Pre comparison (L) and Skin vs Blood comparison (R).

An analysis of miRNA dynamics comparing post-fire to pre-fire activity revealed five miRNAs (miR-125a-3p, miR-548a-5p, miR-1272, miR-362–5p, and miR-421) with significant temporal regulation after multiple testing corrections (q < 0.05) were made (Table 2). Of these miRNAs, miR-125a-3p shows a positive fold change (FC = 1.6), indicating a 60 % increase in expression post-fire activity. The significant upregulation of miR-125–3p suggests the activation of immune (Czimmerer et al., 2016) or stress-related pathways (Nuzziello et al., 2018), highlighting its potential role as a biomarker responsive to external stimuli. Similarly, a differential expression analysis comparing miRNA levels between skin and blood revealed six miRNAs (miR-451a, miR-216a-5p, miR-1286, miR-708-5p, miR-346, miR-421). While other miRNAs show moderate downregulation (FC = 0.73–0.84; 16–27 % reduction), miR-451a is highly downregulated (FC = 0.09), with an miRNA expression level in skin of 9 % of the blood level, corresponding to a 91 % reduction. The consistent downregulation of these miRNAs in skin reflects potential tissue-specific transcription processes or distinct physiological states. There was no significant interaction between sample type and exposure status. The results illustrated in Fig. 1 align with those in Table 2, visually confirming all of these miRNAs except one show significant downregulation (q < 0.05). Only miR-125a-3p was differentially expressed with significant upregulation in post-fire compared to pre-fire activity.

**Fig. 2.**
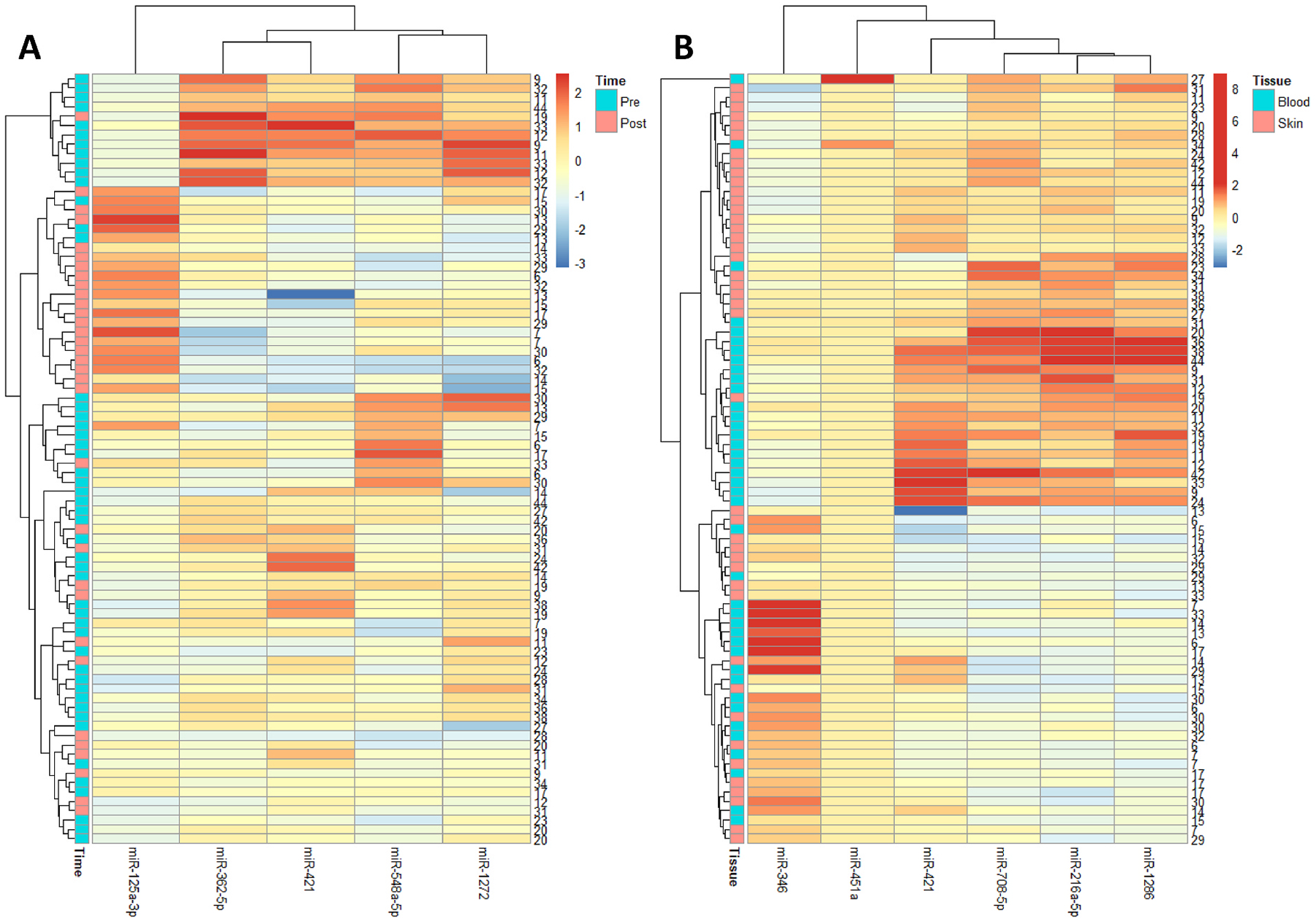
Heatmap of significant miRNAs by fire activity (A) and by biospecimen (B).

A heatmap illustrates the hierarchical clustering of miRNA expression profiles in the collected samples by pre- or post-fire activity (Fig. 2A) and by biospecimen (Fig. 2B). The heatmap confirms that miR-125a-3p is upregulated in multiple post-fire activities, contrasting with its lower expression pre-fire. The rest of the miRNAs show a trend of downregulation in post-fire activities. The distinct clustering patterns and expression changes suggest that exposure to fire smoke during fire activities may trigger specific miRNA-mediated regulatory responses. Based on the differential expression genes by tissue type, many skin and blood samples were clustered in Fig. 2B. Yet, more sub-clusters were observed by biospecimens compared to those by fire activity, suggesting that regulatory responses are influenced by fire activity more than by biospecimen type. This result highlights the potential of miR-125a-3p as a responsive biomarker for environmental stressors such as exposure to fire smoke.

**Fig. 3.**
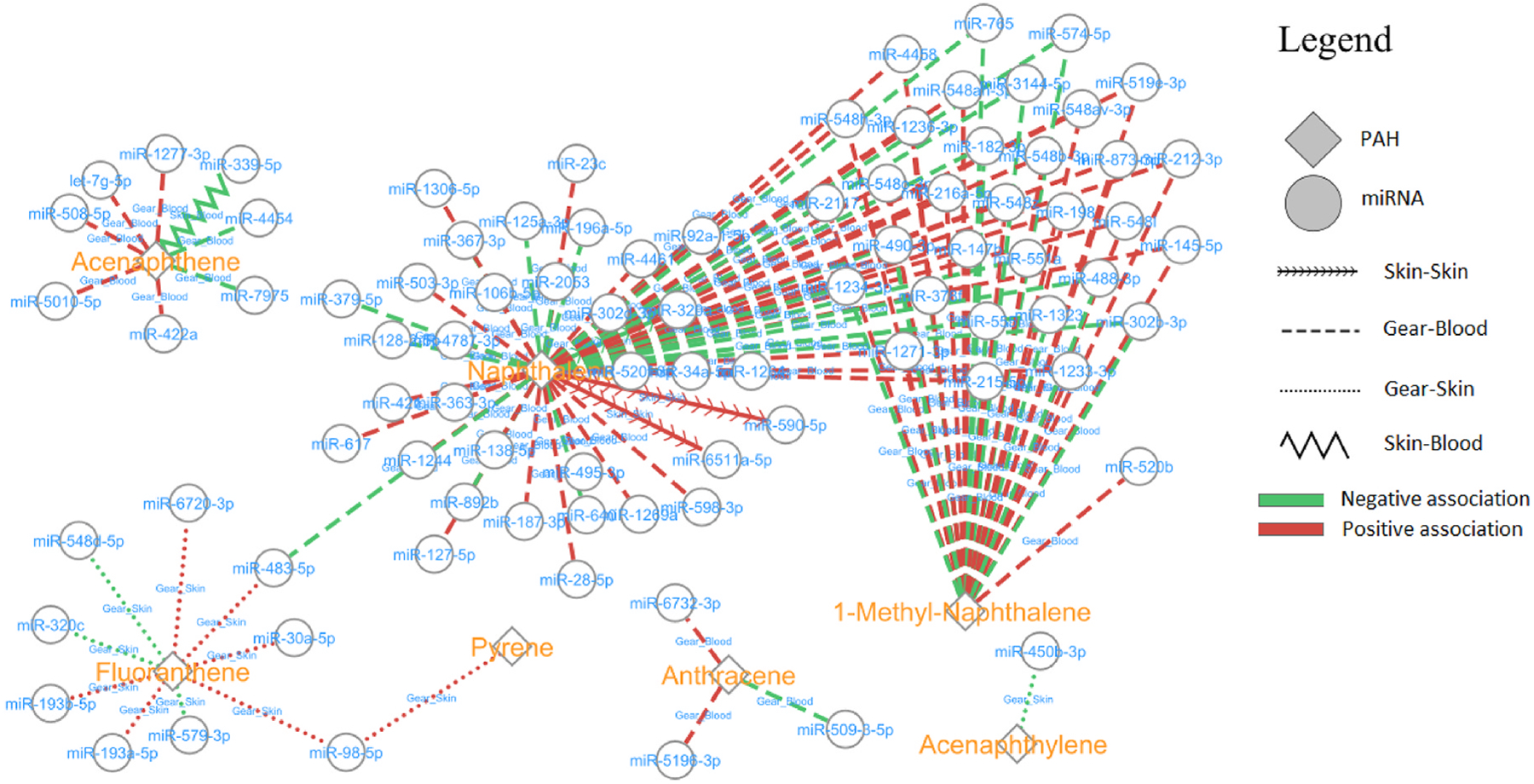
Significant associations between miRNA and PAH in pairs of sample types.

We investigated the interactions between residual PAHs from firefighters’ gear and skin-associated miRNAs in biospecimens using network analysis. A network analysis (Fig. 3) demonstrates the associations between key PAHs (Naphthalene, Acenaphthene, Fluorene, Pyrene, Anthracene, 1-Methyl-Naphthalene, and Acenaphthylene) and the corresponding 106 miRNAs across skin and blood. The complete set of significant associations can be found in Supplementary Table 1. The majority of observed associations (87 %, 92 out of 106) were identified in the interactions between gear-derived PAHs and blood miRNAs. Interestingly, each PAH analyte shows a distinct pathway, for example, 1-Methyl-Naphthalene shows a Gear-Blood pathway and Fluoranthene, a Gear-Skin pathway. Naphthalene emerges as the most central PAH, displaying extensive associations with a wide array of miRNAs. Naphthalene and 1-Methyl-Naphthalene show the most miRNA interactions (except for one in 1-Methyl-Naphthalene), albeit with the strongest pathway being Gear-Blood for most positive and negative associations, a finding consistent with Table 2.

**Fig. 4.**
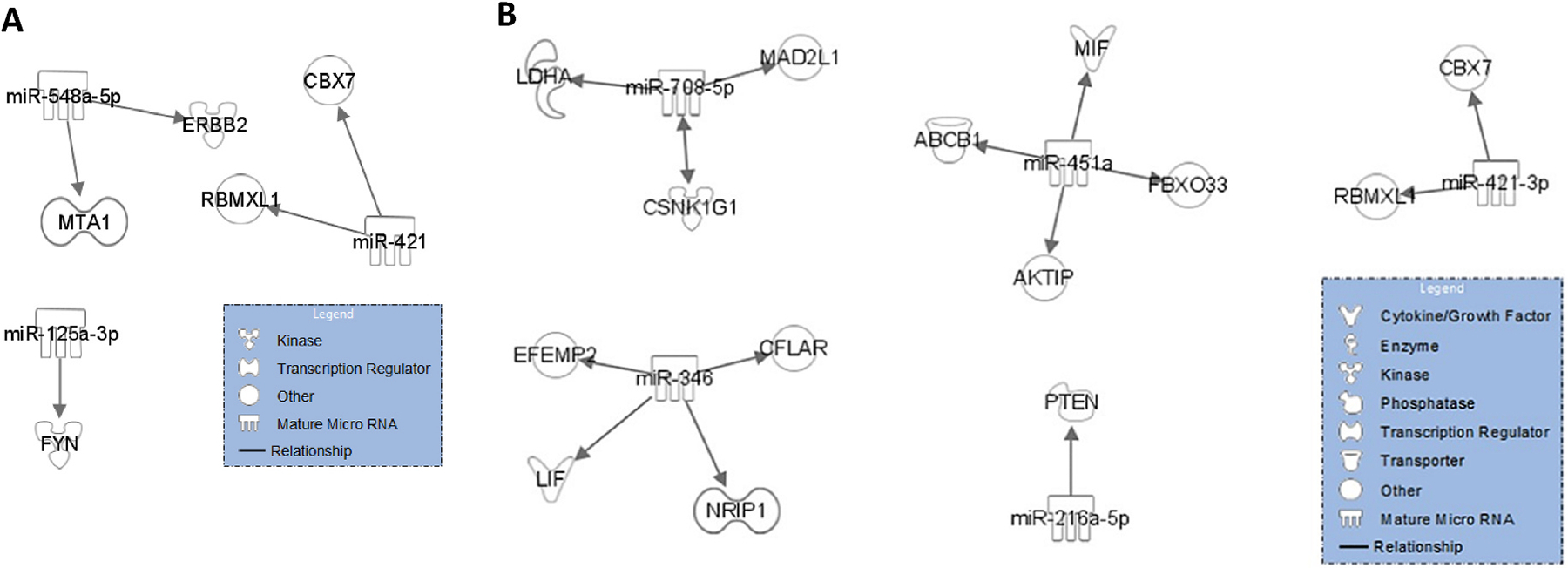
Functions of the significant miRNAs and their target messenger RNAs (mRNAs) by fire activity (A) and by biospecimen (B).

We further examined the functions of the significant miRNAs and their target messenger RNAs (mRNAs) using gene set enrichment analysis and annotation through the use of IPA. The downstream bioinformatics analyses reveals several experimentally validated target mRNAs known as biomarkers for multiple diseases. Specifically, we find five (ERBB2, FYN, MTA1, CBX7, RBMXL1) experimentally validated target mRNAs targeted by three of the five significant miRNAs in post-fire activity compare to pre-fire activity (Fig. 4A and Table 3). ERBB2, a kinase located in plasma membrane, encodes a member of the epidermal growth factor (EGF) receptor family of receptor tyrosine kinases. ERBB2 is a well-known diagnostic, prognostic, or efficacy biomarker for many cancer types, such as brain cancer, colorectal cancer, and lung cancer. FYN, a member of the protein-tyrosine kinase oncogene family, encodes a membrane-associated tyrosine kinase that has been implicated in the control of cell growth. The MTA1 gene encodes a protein that has been identified in a screen for genes expressed in metastatic cells, specifically, mammary adenocarcinoma cell lines. Expression of this gene has been correlated with the metastatic potential of at least two types of carcinomas, although it is also expressed in many normal tissues. Similarly, we found 14 experimentally validated target mRNAs targeted by five of the six significant miRNAs in skin compared to blood (Fig. 4B and Table 3). For example, ABCB1 encodes a membrane-associated protein, which is a member of the superfamily of ATP-binding cassette (ABC) transporters. ABCB1 is a prognostic or efficacy biomarker for multiple diseases, such as HIV infection, acute myeloid leukemia, and melanoma. MIF encodes a lymphokine involved in cell-mediated immunity, immunoregulation, and inflammation, playing a role in the regulation of macrophage function in host defense through the suppression of anti-inflammatory effects of glucocorticoids. MIF has been used as a biomarker for the diagnosis of ovarian cancer and pulmonary hypertension and for the prognosis of acute respiratory distress syndrome and sarcoma. CBX7 and RBMXL1, which are common targeted genes across fire activity and biospecimens, both regulate gene expression. CBX7 controls chromatin remodeling and gene silencing, while RBMXL1 is involved in RNA splicing. Both proteins ensure proper gene regulation at different stages, with CBX7 acting at the transcriptional level and RBMXL1 at the post-transcriptional level.

**Fig. 5.**
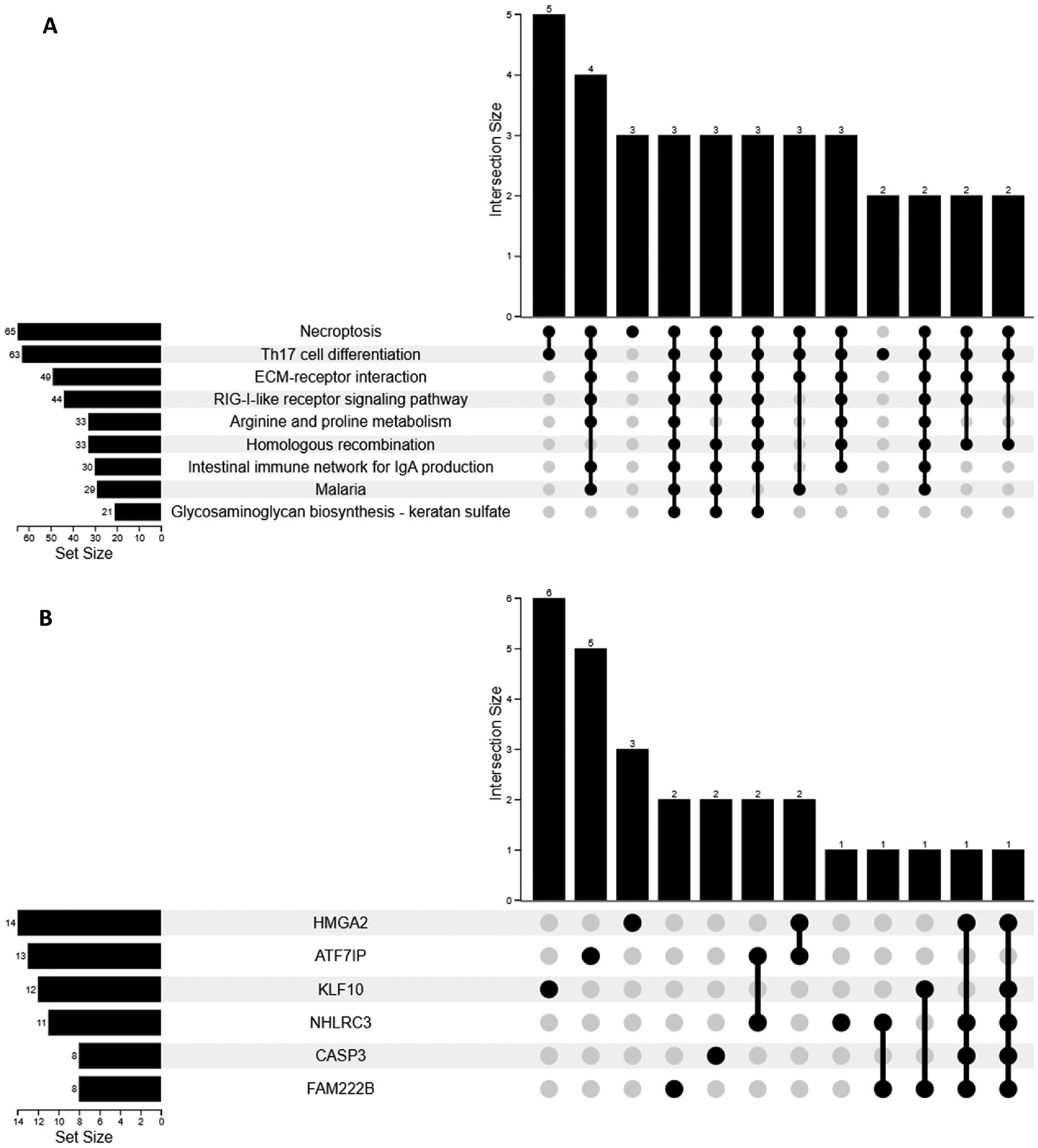
Upset plots of significantly enriched pathways (A) and genes (B) based on 106 miRNAs. Both plots illustrate the intersection of enriched pathways (vertical bars) and individual pathway sizes (horizontal bars), highlighting both unique and overlapping miRNA-associated regulatory networks.

A pathway enrichment analysis using miEAA (Aparicio-Puerta et al., 2023) identified nine significantly enriched KEGG pathways (Fig. 5A) and six significantly enriched target genes (Fig. 5B) in our miRNA-targeted gene set. In particular, CASP3 is a known diagnostic and prognostic biomarker for breast cancer, bladder cancer, and pancreatic cancer (Karam et al., 2007; Z. Zhou et al., 2022). An analysis of pathway intersections revealed significant gene overlap between necroptosis and Th17 cell differentiation pathways. The matrix visualization (lower portion) indicates that several genes participate in multiple biological pathways, particularly between cell death regulation and immune response processes. These results suggest coordinated cellular mechanisms involving both programmed cell death and immune regulation in our study participants, with potential functional crosstalk between these biological processes. Similarly, Fig. 5B illustrates the intersection of gene sets, identifying shared and unique gene associations among six genes (*HMGA2, ATF7IP, KLF10, NHLRC3, CASP3*, and *FAM222B)*. *HMGA2* exhibits the largest set size (n = 14) and the most significant intersection, involving six elements, is shared between *HMGA2* and *ATF7IP*, suggesting a strong co-occurrence between these genes. FAM22B exhibits the most complex interaction profile despite having the smallest set size, with connections at positions 4, 7, 10, 11, and 12. This result suggests FAM22B functions as a central regulatory hub integrating signals from multiple pathways.

## 4. Discussion

We quantified exposure to PAH on firefighter’s turnout gear and skin, then integrated an analysis of miRNA expression changes in dermal and blood samples from the firefighters. This systematic approach advances our understanding of the impact of environmental exposures at an emergency fireground on firefighters’ miRNA transcripts. Our discussion focuses on a set of significant differential expressions of miRNAs and their roles in PAH-associated human cancers and diseases. Specifically, we discuss the alteration of miRNAs in firefighters due to PAH exposure, network of PAH exposure-miRNA interactions, their oncogenic or tumor-suppressive functions in various cancers by fire activity and by biospecimen, and our recommendations including potential utility as biomarkers of PAH exposure and cancer risk.

### 4.1. PAHs-associated miRNAs

Polycyclic aromatic hydrocarbons (PAHs) are major organic byproducts generated through incomplete combustion processes during fires. Fire smoke, produced as firefighters work to suppress fires, contains a range of toxic chemical compounds, among which PAHs are considered particularly hazardous due to their persistence and potential health risks (ASTDR, 2015; Hwang et al., 2021; U.S. EPA, 2022). As far as we know, our network is the first to reveal a distinct topology with seven PAH analytes from fire smoke that interacted with 106 miRNAs (Fig. 3). The PAHs in this network, specifically Naphthalene, represent a class of possibly carcinogenic to humans (Group 2B) toxins while the rest of the PAHs are not classifiable (Group 3) as the level of evidence of carcinogenicity is insufficient (IARC, 2024). The network visualization demonstrates two primary interaction clusters between Naphthalene and 1-Methyl-anthracene, with one exception – miR-520b. We also found that PAHs primarily upregulate miRNA expression. These positive associations suggest that PAHs may contribute to disease development through miRNA upregulation, potentially affecting downstream gene expression patterns. This positive pattern aligns with previous findings that PAHs such as benzo[a]anthracene and benzo[k]fluoranthene can significantly upregulate miRNA expression, particularly miR-181 family members in hepatocellular carcinoma cells (Song et al., 2013). Similarly, benzo[a]pyrene can upregulate p53-targeting miRNAs, including miR-25, miR-92, miR-141, and miR-200a in multiple myeloma cells, as PAHs activate the aryl hydrocarbon receptor (AhR)(Gordon et al., 2015; Rude et al., 2024). In contrast, Acenaphthylene showed a negative interaction with a specific miRNA (miR-450b–3b). These distinct regulatory signatures suggest analyte-specific mechanisms of action, supporting previous findings that different PAHs can activate distinct molecular pathways leading to varied toxicological outcomes(Mallah et al., 2022).

### 4.2. Network analysis and PAH-miRNA pathways

Among the exposure-expression network combinations evaluated, PAHs detected on firefighting turnout gear showed the strongest associations with blood miRNA expression. Significant correlations were observed specifically for low molecular weight (LMW) PAHs, including acenaphthene, anthracene, naphthalene, and 1-methyl-naphthalene. LMW PAHs generally are greater bioavailability due to their higher volatility and lower hydrophobicity that enable rapid skin and respiratory absorption(Patel et al., 2020). These results suggest that dermal contact or inhalation of LMW PAHs from contaminated gear may induce systemic molecular alterations reflected in circulating miRNA profiles. However, we were not able to distinguish between the exposure routes of dermal absorption and inhalation of the PAHs from this study. The marked downregulation in skin tissue suggests that significant differentially expressed miRNA may participate in blood-specific regulatory circuits or act as suppressors of skin-related pathways. Thus, deposited PAH toxins on gear mechanisms may differ across biospecimen interfaces, with particularly pronounced effects of miRNA circulating in blood. This finding correlates with our earlier finding that exposure to PAHs on gear is more sensitive than on skin, and primarily transported via the blood(Zhu et al., 2024). Yet, whether the levels of miRNA in biospecimens are related to occupational factors, including detailed turnout gear practices and PAH exposure assessments, is still largely undetermined. The rest of the PAHs display distinct associations within miRNAs, suggesting analyte-specific miRNA regulatory mechanisms.

Pyrene, Anthracene, and Acenaphthylene show fewer miRNA associations, suggesting more localized or restricted biological interactions. A negative Skin-Blood pathway association is found with Acenaphthene, suggesting potential miRNA-mediated suppression mechanisms in response to exposure to Acenaphthene.

### 4.3. Functions of significant miRNAs

The simultaneous enrichment of both necroptosis and Th17 differentiation pathways suggests a potential interplay between programmed cell death mechanisms and immune regulation in our study, which warrants further investigation. Necroptosis appears as the most significantly enriched pathway, with five miRNA-target genes contributing to its enrichment. This genetically regulated form of necrotic cell death is emerging as an important pathogenic mediator in various human diseases(Choi et al., 2019). The strong enrichment of necroptosis suggests that regulated cell death mechanisms play a crucial role in the biological processes under investigation, potentially indicating cellular stress, inflammatory responses, or tissue injury mechanisms. Th17 cell differentiation represents the second most enriched pathway. Th17 cells are a subset of CD4^+^ T cells that secrete IL-17 and play critical roles in immune regulation, host defense, and autoimmune diseases(Yasuda et al., 2019). The significant enrichment of this pathway suggests active immune regulatory processes in our study participants, potentially indicating inflammatory responses or immune system activation(Graeber and Olsen, 2012).

#### By fire activity:

Several studies have demonstrated that firefighting exposure is associated with significant changes in both miRNA expression patterns and miRNA enrichment related to cancer pathways, including carcinoma, hepatocellular carcinoma, and thyroid neoplasms (Jeong et al., 2018; Jung et al., 2021). In this study, we found several miRNAs to be significantly differentially expressed by fire activity, with miR-125a-3p the only miRNA observed to be upregulated post-fire. miR-125a-3p has been found to inhibit chemo resistance and induce apoptosis in prostate cancer cells by targeting MTA1, supporting its role as a tumor suppressor in this context (Liu et al., 2017). Additionally, other studies have demonstrated that miR-125a-3p is downregulated in murine models of silica-induced fibrosis, which its overexpression has been shown to alleviate. miR-125a-3p is expressed at higher levels in normal lung tissue than in tumors (Hou et al., 2017) and plays a crucial role in pulmonary fibrosis by regulating fibroblast activation through the FYN/STAT3 signaling pathway (Xu et al., 2019). While miR-125a-3p is typically downregulated in tumor tissues, one study found that circulating exosomal miR-125a-3p is upregulated in plasma of early-stage colon cancer patients (Wang et al., 2017). This apparent contradiction highlights the complex nature of miRNA regulation and suggests different mechanisms at play between tissue expression and release into circulation.

#### By biospecimen:

Another set of miRNAs were significantly differentially expressed by biospecimen. miR-216a-5p, for instance, reduces renal injury by targeting PTEN, an important protein, thereby regulating apoptosis through the Akt pathway(Zhang et al., 2020). A study on breast cancer progression also discovered that miR-216a-5p interacts with PTEN, which is known to suppress tumors(Wu et al., 2021). A similar role of miR-216a-5p has been examined in cardiac fibroblast function and fibrosis. Specifically, miR-216a-5p downregulates PTEN, activating the Akt pathway, which promotes proliferation and fibrogenesis in cardiac fibroblasts and contributes to cardiac fibrosis(Tao et al., 2019). Overall, miR-216a-5p negatively regulates **PTEN** expression, demonstrating its critical role in influencing disease processes through PTEN downregulation. The miR-216a-5p/PTEN axis is identified as a potential therapeutic target in all three conditions, as modulating this pathway could mitigate disease progression by restoring PTEN levels or blocking the associated signaling pathways.

#### By fire activity and biospecimen:

Only miR-421 was an miRNA common to both fire activity and biospecimen. The majority of studies characterize miR-421 as an oncogene. miR-421 promotes tumor growth and survival by repressing tumor suppressor targets. Elevated miR-421 levels in prostate cancer patients are linked to higher Gleason scores, advanced tumor stage, and presence of metastasis(Huang et al., 2025). miR-421 also plays a role in the tumor microenvironment. Cancer-associated fibroblasts secrete exosomal miR-421, which is taken up by pancreatic cancer cells and accelerates tumor progression(B. Zhou et al., 2022). Unlike the above cancers, breast cancer exhibits *downregulation* of miR-421. miR-421 is significantly low expressed in breast tumors and metastatic breast cell lines(Pan et al., 2016).

### 4.4. Practical guidelines and recommendations

The Firefighter Cancer Registry Act of 2018, enacted by the U.S. Congress, requires the Centers for Disease Control and Prevention (CDC) to develop and maintain a voluntary cancer registry, called the National Firefighter Registry for Cancer (NFR), focused on cancer incidence among firefighters(U.S. Congress, 2018). This ongoing project implies not only that the federal government recognizes the significant scope of research needed but also that scientific efforts to address the unique challenges of firefighters should be continued. Despite being a pilot, our study indicates that miRNA-based biomarker research could strongly complement the NFR. First, we recommend conducting longitudinal cohort studies to monitor firefighters throughout their professional careers, assessing both occupational exposures and biomarkers – miRNA expression dynamics – over time. Integrating NFR data with these findings could enable the development of personalized cancer risk assessments, facilitating earlier interventions such as enhanced screening protocols for firefighters identified with high-risk miRNA profiles.

Second, an occupational epidemiological study could effectively reconstruct comprehensive exposures by studying biomarkers such as miRNA expression levels and pathways. miRNA research in the biomedical and clinical research areas has exponentially increased over the past decade due to the fast development and commercial accessibility of the needed technology. miRNAs are important mediators and post-transcriptional regulators of expression because of their ability to degrade and/or suppress the translation of multiple mRNA molecules (Guo et al., 2010). Genetically and epigenetically altered miRNA, which is involved in carcinogenesis and metastasis, is associated with either oncogenes or tumor suppressor genes in cancer(Jeong et al., 2018; Li et al., 2018). Thus, miRNA provide crucial information for fully understanding cancer pathways and cancer etiology. The potential interaction between miRNAs and the pathways involved in cancer development can be used to integrate miRNA expression profiles(Jeong et al., 2016).

Furthermore, incorporating individual miRNA profiling in biomarker patterns with occupational exposure measurements will significantly reduce misclassification errors, resulting in better estimates for constructing individual job exposure matrices (JEMs) specific to this unique cohort group. Traditionally, a JEM is created using the identified health risks associated with exposure data (e.g., either primary measurements or a secondary database) and surrogate information (e.g., work history, job title). Collectively, direct measuring a panel of carcinogens (e.g., PAHs)-responsive miRNAs in accessible biospecimens (e. g., blood, skin) could enable early detection of high-risk firefighters and monitoring of intervention efficacy(Sisto et al., 2019). Furthermore, some of these miRNAs might be explored as therapeutic targets – for example, restoring a PAH-suppressed miRNA (like miR-216a-5p) in tumors or inhibiting an oncogenic miRNA (like miR-421) might reverse certain molecular damage of PAHs.

This study is the first to systematically investigate and quantify the mechanism by which PAHs on turnout gear and skin can alter miRNA expression, modulating its potential impact on carcinogenesis and addressing a critical gap in the literature. Given the small window of opportunity at a fire activity, short-term effects were explored by comparing pre- and post-exposure levels with unique work characteristics, including respirator practices. A recent study on wildland firefighters found that 65 miRNAs significantly changed between pre- and post-wildfire season samples, suggesting miRNA expression is a responsive epigenetic marker of short-term smoke exposure (Goodrich et al., 2025). Thus, the significance of this study is based on the scientific premise that specific targeted exposure to PAHs at pre- and post-fire activities allowed us to more accurately and reliably measure the alteration of miRNA expression and identify early indicators of the carcinogenic process. In summary, miRNAs act as critical mediators linking environmental carcinogen exposure to human cancer development. Fireground PAH exposure induces specific miRNA expression changes in firefighters, with consistent patterns observed across skin and blood biospecimens. These miRNAs target gene networks implicated in occupational carcinogenesis and immune modulation, offering promising biomarkers for occupational health surveillance. Thus, identifying exposure-response biomarkers may enhance risk prediction and support the development of effective intervention and prevention strategies.

### 4.5. Strengths and limitations

miRNAs are both abundant, as they are found in red blood cells and extracellular vesicles (Woeller et al., 2016). While their half-lives vary, miRNAs demonstrate remarkable stability in blood once released into circulation due to their small size and presence in protein complexes. The experimental results found that the half-lives of miRNA were approximately 16.42 ± 4.2 h in blood (Wang and Liu, 2022). Thus, we collected blood-circulating miRNAs for assessing the health effects. Also, the skin tape strip (STS) is a valid sampling method and an adequate alternative to a whole skin biopsy, having been used for transcriptome gene expression analysis in clinical and pathological diagnostics(Dyjack et al., 2018; Kim et al., 2019). To our knowledge, this is the first miRNA study to investigate both blood and skin in an occupational cohort of firefighters. Despite its contributions, this study has limitations that warrant consideration. We determined that restricting our study to PAHs of all the complex toxic compounds present in fire smoke was our best approach. Simultaneously, assessing biological responses using miRNA oversimplifies the complex exposure to fire smoke that firefighters encounter. Thus, the correlation between PAHs and associated microRNAs in firefighter biospecimens should not be interpreted as evidence of causation. Our study may represent preliminary evidence on the association of accumulated PAH and miRNA expression. Although we did not include experimental validation to support bioinformatic analyses, we acknowledge its absence as a limitation. Nonetheless, our findings provide an initial framework for identifying candidate miRNAs responsive to fire-related carcinogen exposure, and lay the foundation for future studies. Another key limitation of this study is the relatively small sample size, which may limit the generalizability of the findings. Additionally, the cross-sectional design precludes establishing causal relationships between exposures and miRNA expression changes, highlighting the need for longitudinal studies to confirm these associations. Specifically, carcinogenic exposures, including those from PAHs, should be examined using longitudinal studies because miRNA alterations from reversible to irreversible depend on both dosage and duration of exposure (Jeong et al., 2018; Li et al., 2018). Initially, the field-exposure assessment was designed to measure PAH levels and biospecimens via repeated measurements. Yet, the study period (Dec. 2021–Jun. 2022) was harshly affected by the pandemic, so the original approach was not feasible. Thus, there was no record of repeated samples from the same participants nor the exact timing of the sampling within the 24-h window in this study. While the baseline for pre-fire activities was established using pre-exposure sampling at the fire departments, the post-sampling campaigns were conducted within 24 h after the fire suppression, but before the gear was washed. Initially, the post-fire sampling at the fire scene was designed so that the firefighters immediately doffed the outer layers of their gear at the scene while leaving the inner layers on for the rest of the work shift or longer(Wingfors et al., 2018), but this approach was challenging during the pandemic. We also were not able to monitor physical activity and stress after each fire activity. Finally, the severe thermal conditions and moist environment during fire extinguishment, in conjunction with the up-to-35 kg of multi-layered turnout gear worn by firefighters, leads to acute physical activity with a higher core heat stress, which may trigger epigenetic regulation including changes in miRNA expression.

## Supplementary Material

1

## Figures and Tables

**Table 1:** Demographic characteristics of study participants by Firefighter group: Career and volunteer.

Characteristics	Total (N = 25)	Career (N = 12)	Volunteer (N = 13)	P-value
**Age**				
Mean (SD)	39 (±12)	36 (±9.9)	41 (±13)	0.414^b^
**Gender**				
Male	23 (92.0 %)	11 (91.7 %)	12 (92.3 %)	>0.999^c^
Female	2 (8.0 %)	1 (8.3 %)	1 (7.7 %)	
**Race**				
Non-Hispanic White	24 (96.0 %)	12 (100 %)	12 (92.3 %)	>0.999^c^
Hispanic Native	1 (4.0 %)	0 (0 %)	1 (7.7 %)	
**Height, meter**				
Mean (SD)	1.8 (±0.080)	1.8 (±0.073)	1.8 (±0.089)	0.868
**Weight, kg**				
Mean (SD)	94 (±17)	89 (±13)	99 (±18)	0.117
**Years served**				
Mean (SD)^a^	12 (±9.3)	12(±9.5)	12 (±9.4)	0.908^b^

a The data is complete due to a participant’s non-response.

b Wilcoxon rank test when the variable could not retain null hypothesis of Shapiro Normality test.

c Fisher’s exact test.

**Table 2:** Significant Differential Expression of miRNAs by Fire activity and by Biospecimen.

Name	Accession	Fold Change	95 % CI		p-value	q-value^a^
			Lower	Upper		
**Fire Activity (Post vs. Pre)**						
miR-125a-3p	MIMAT0004602	1.60	1.32	1.94	1.73E-06	0.001
miR-548a-5p	MIMAT0004803	0.77	0.68	0.88	5.49E-05	0.020
miR-1272	MIMAT0005925	0.78	0.69	0.88	7.39E-05	0.020
miR-362–5p	MIMAT0000705	0.78	0.68	0.89	1.79E-04	0.036
miR-421	MIMAT0003339	0.83	0.75	0.92	3.69E-04	0.039
**Biospecimen (Skin vs. Blood)**						
miR-451a	MIMAT0001631	0.09	0.05	0.16	3.61E-12	2.77E-09
miR-216a-5p	MIMAT0000273	0.75	0.67	0.84	8.36E-07	3.21E-04
miR-1286	MIMAT0005877	0.73	0.64	0.84	5.01E-06	0.001
miR-708-5p	MIMAT0000085	0.78	0.70	0.88	3.99E-05	0.008
miR-346	MIMAT0000773	0.78	0.69	0.88	7.05E-05	0.011
miR-421	MIMAT0003339	0.84	0.76	0.92	2.76E-04	0.035

a All q-values fall <0.05 false discovery rate (FDR) threshold, indicating <5 % of statistically significant results in this analysis are likely false positives.

**Table 3:** Target Genes of Significant miRNA.

Symbol	Entrez Gene Name	Location	Type(s)	Biomarker Application(s)	Biomarker-Related Disease
**Fire Activity (Post vs. Pre)**					
ERBB2	erb-b2 receptor tyrosine kinase 2	Plasma Membrane	kinase	diagnosis, disease progression, efficacy, prognosis, safety, unspecified application	lung cancer, breast cancer, endometrial carcinoma, pancreatic cancer, osteosarcoma, colorectal cancer, head and neck cancer
FYN	FYN proto-oncogene, Src family tyrosine kinase	Plasma Membrane	kinase	efficacy	prostate cancer
MTA1	metastasis associated 1	Nucleus	transcription regulator	diagnosis, response to therapy	breast cancer, prostate cancer, endometrial cancer
**Biospecimen (Skin vs. Blood)**					
ABCB1	ATP binding cassette subfamily B member 1	Plasma Membrane	transporter		
AKTIP	AKT interacting protein	Cytoplasm	other		
CFLAR	CASP8 and FADD like apoptosis regulator	Cytoplasm	other	prognosis, unspecified application	Burkitt’s lymphoma, Huntington’s disease
CSNK1G1	casein kinase 1 gamma 1	Cytoplasm	kinase		
EFEMP2	EGF containing fibulin extracellular matrix protein 2	Extracellular Space	other		
FBXO33	F-box protein 33	Other	other		
LDHA	lactate dehydrogenase A	Cytoplasm	enzyme	efficacy, unspecified application	lung cancer, Huntington’s disease
LIF	LIF interleukin 6 family cytokine	Extracellular Space	cytokine	unspecified application	Sjogren’s syndrome
MAD2L1	mitotic arrest deficient 2 like 1	Nucleus	other		
MIF	macrophage migration inhibitory factor	Extracellular Space	cytokine	diagnosis, prognosis, response to therapy	pulmonary hypertension, ovarian cancer, sarcoma, acute respiratory distress syndrome
NRIP1	nuclear receptor interacting protein 1	Nucleus	transcription regulator		
PTEN	phosphatase and tensin homolog	Cytoplasm	phosphatase	diagnosis, disease progression	colorectal cancer, bladder cancer, breast cancer
**Both Fire activity and Biospecimen**					
CBX7	chromobox 7	Nucleus	other		
RBMXL1	RBMX like 1	Nucleus	other		

## Data Availability

The datasets generated and analysed during this study will be available during the online submission process.

## Appendix A. Supplementary data

Supplementary data to this article can be found online at https://doi.org/10.1016/j.envres.2025.122348.
